# Nutritional, Physicochemical, and Organoleptic Properties of Camel Meat Burger Incorporating Unpollinated Barhi Date Fruit Pulp

**DOI:** 10.1155/2022/4581821

**Published:** 2022-10-15

**Authors:** El Sayed, A. Abd El-Hady, Hani A. Alfheeaid, Mohamed M. Abd El-Razik, Sami A. Althwab, Hassan Barakat

**Affiliations:** ^1^Department of Food Science and Human Nutrition, College of Agriculture and Veterinary Medicine, Qassim University, Buraydah 51452, Saudi Arabia; ^2^Hotel Studies Department, Faculty of Tourism and Hotels, Suez Canal Univ., Ismailia, Egypt; ^3^Food Science Department, Faculty of Agriculture, Ain Shams University, Cairo, Egypt; ^4^Food Technology Department, Faculty of Agriculture, Benha University, Moshtohor 13736, Egypt

## Abstract

The chemical composition of unpollinated Barhi date fruit (UBDF) (at khalal maturity stage) pulp and its effect as fiber source or fat replacer on the quality characteristics of camel meat burgers were investigated. The UBDF was characterized by high total carbohydrate, fiber, and mineral contents. It contains substantial amounts of fiber (19.60%) and low-fat content. Prepared UBDF pulp was added to camel meat burger as a fiber source at 2.5, 5, 7.5, 10, and 15% instead of camel meat and as a fat replacer at 50, 75, and 100% instead of camel-back fat during manufacturing of camel meat burgers. Chemical composition, mineral content, instrumental color, cooking characteristics, and organoleptic properties were evaluated. Results indicated that adding UBDF as fiber source significantly reduced moisture and protein contents and increased total carbohydrates, including fiber content. In camel meat burgers, K and Ca significantly increased in UBDF level-dependent manure. Adding UBDF as a fat replacer significantly increased moisture and total carbohydrate contents, while a significant reduction in fat content has been remarked. Increasing the levels of UBDF pulp as either fiber source or fat replacer in camel burgers improved (*p* < 0.05) shrinkage, cooking loss, and cooking yield in roasted camel burger level-dependent manure. The incorporation of UBDF pulp in camel burgers increased the lightness (*L*^∗^) values and decreased the redness (*a*^∗^) and yellowness (*b*^∗^) significantly. The formulated camel burgers with different UBDF pulp levels revealed better organoleptic characteristics than normal camel meat burgers. Interestingly, adding UBDF as fiber source at 7.5-15% presented overall acceptability of more than 90% compared to the control sample of 81.7%. In the same context, adding UBDF as a fat replacer at 100% replacing the level of added fats scored overall acceptability of more than 93.60% compared to the control sample of 67.4%. Generally, according to the results of this study, it could be concluded that UBDF pulp could be used as a functional additive to produce high-fiber and/or low-fat camel meat burgers.

## 1. Introduction

Consumers are more concerned about their health and the fat content of the foods they consume. Most shoppers nowadays try to keep up their health by eating balanced meals, especially as more people become aware of the links between what they eat and how they feel. Fiber is a common ingredient in healthful diets because of its beneficial effects on digestive health [1, 2]. Consuming enough fiber has been linked to a reduced risk of various diseases, including cancer, obesity, and cardiovascular disease [3, 4]. Recent years have seen a rise in the status of fruits and vegetables as a solution for obtaining physiologically active ingredients like fiber [5].

Meat and its products comprise a significant portion of the average human's daily caloric intake. They provide protein, amino acids, lipids, vitamins, and minerals [6]. Despite these positive attributes, they are frequently viewed unfavorably due to their high quantities of saturated fats, cholesterol, salt, nitrite, and lack of nondigestible carbohydrates [7–9]. Some negative aspects could be reduced using lean meat cuts, which eliminate fats and cholesterol [10]. Fibers, as a functional element, can also be added to meat products to boost their perceived healthfulness. Since fibers serve multiple purposes in the food industry, including as an extender, binder, and fat replacement, they are frequently used to prepare various meat products [11]. It is economically beneficial for customers and processors to employ fibers as a bulking agent in beef products because they improve texture, reduce cooking loss, and increase water binding and fat retention [12–14]. Applying dietary fibers to produce frankfurters, dry fermented sausages, and beef patties was investigated [13, 15].

Camels (*Camelus dromedarius*) are one of the most important sources of meat in the desert since they can withstand extreme temperatures and water scarcity [16]. The world population of dromedary camels is estimated to be 35.5 million, mostly in the Arab world. Saudi Arabia (SA) has roughly 490000 heads (FAOSTAT, [17]). The meat of young camels, less than three years of age, is comparable in taste and texture to beef [18]. Camel meat has high health and nutritional aspects as it has a high content of protein (17.0-23.7%), low fat content (1.1-6.2%), high content of polyunsaturated fatty acids, and low cholesterol content [19–21]. In recent years, camel meat has become a popular alternative to other red meats [22]. Camel meat can be processed into goods generally made from beef and other red meats [23–25]. Global processed camel meat production data is unavailable. Many meat products such as patties, sausages, burgers, and shawarma may be made from camel meat, increasing its use and popular acceptability [19, 26]. Dawood [27] made camel burgers from camel chuck at 0, 5, and 10% fat levels using a basic formulation. Ibrahim and Nour [28] prepared burgers from beef replaced at five levels with camel meat (0, 25, 50, 75, and 100%). Results showed that camel meat boosted burger softness, flavor, juiciness, and color. Also, sensory panelists preferred camel burgers with 10% fat and 6% soybean husks, according to Al-khalifa and Atia [29]. Plant dietary fibers can be used in meat products to improve cooking and color properties [30, 31]. Color characteristics (hue, chroma, and value) of meat products were affected by fiber addition. The utilization of dietary fiber as fat replacers in meat products improved the visual color of produced meat products [32–34]. Kurt and Kılınççeker [35] reported that adding fibers to meatballs boosted product quality and improved color properties.

The date palm (*Phoenix dactylifera* L.) is one of the most important crops in SA. The latest statistics from the General Authority for Statistics [36] showed that the total number of palm trees in the Kingdom amounted to 31,234,155 palm trees. Qassim came in the second position with 7,542,914 palm trees, 24% of SA's total trees. The National Center for Palms and Dates (2020) reported that the number of Barhi palm trees (1534671) represents about 5% of the total palm trees in SA, indicating the importance of this type for the agricultural economy in SA and in particular Qassim region. Due to annual increases in date losses in SA, date wastage and loss for 2019 reached 21.5% presenting 137000 tons [36]. This led to developing food products with unmarketable dates or overstock from prior years. Barhi dates are commonly farmed in Qassim. Unpollinated dates resulting from the failure of fertilization for many palm blossoms constitute around 15% of the wastage of dates on farms, making it a huge concern for farmers and producers of dates and their marketing or fresh consumption acceptability. A study conducted by El-Habbab et al. [37] reported the loss of dates at harvest time for three types of dates in SA. He examined the proportion of unpollinated fruits and bisrs developed due to a pollination fault. Due to these variables, regional variants vary. Unpollinated fruits yield little, inferior fruits with no kernel, unlike the one called “Shees.” These unpollinated, high-fiber fruits are termed “Alhashf.” This wastage fiber could be utilized in the food industry to modify the properties of dairy, meat, bread, and jam products [38]. UBDF is a unique source of dietary fiber to generate low-fat or high-fiber functional foods that benefit community health and SA palm producers. Therefore, this study is aimed at investigating the potential use of unpollinated Barhi date fruit (UBDF) pulp produced from dry unpollinated Barhi date fruits during the khalal stage, known for their high fiber content in the preparation of low-fat and high-fiber camel meat burgers.

## 2. Materials and Methods

### 2.1. Materials

Fresh boneless camel meat (Longissimus dorsi muscles) and camel-back fat were purchased from a local market in Qassim, SA. UBDF at the khalal stage were obtained from a farm in Qassim. Corn starch, salt, cumin, onion, garlic, and black pepper were purchased from a local market.

### 2.2. Preparation of UBDF Pulp

UBDF pulp was from the raceme, sorted, washed thoroughly with water, crushed (TA149D, Tagliaverdure Universal, Italy), and then blanched for 5 min at 95°C. The blanched paste was homogenized to obtain fine fruit pulp and then stored at −18 ± 1°C for further use. The resultant pulp was used in preparing camel meat burgers as a novel source of dietary fiber or as a fat replacer.

### 2.3. Preparation of Camel Meat Burgers

Fresh boneless camel meat was chopped into small pieces after removing visible fat and connective tissues. Using a meat mincer (Bizerba, Wilhelm Kraut GmbH & Co. KG, Germany), chopped meat samples and camel-back fat were minced separately using a plate with 4 mm holes. The minced camel meat was mixed with fat, date fruit pulp, starch, water, and spices (a mixture containing 2.0 g salt, 0.4 g cumin, 3.0 g onion, 0.5 g garlic, 1.0 g black pepper, and 0.5 g spice mixture) according to percentages shown in Table 1. Different ingredients in each blend were homogenously mixed for 5 min in a Classic Chef-KM353 mixer (Kenwood Ltd., Havant, U.K.). After mixing, burgers of 50 ± 1 g were formed using a burger-forming device (Italians, Italy). The resultant burgers were placed on Styrofoam trays, wrapped with polyethylene film, and kept in the refrigerator at 4 ± 1°C until analysis. Different prepared camel meat burger samples were roasted to determine the effects of adding UBDF pulp as a fiber source or fat replacer on the resultant burgers' color parameters and sensory attributes.

### 2.4. Proximate Analysis and Mineral Determinations

The moisture (method no. 925.10), crude protein (N x 6.25) (method no. 978.04), crude fat (method no. 930.09), and ash (method no. 930.05) contents were determined according to the guidelines of the Association of Official Analytical Chemists [39]. Carbohydrate content was calculated by difference. Crude fibers were estimated according to Ajila et al. [40]. The mineral contents of camel meat, UBDF pulp, and the resultant burger samples, including potassium, calcium, zinc, iron, magnesium, copper, and cobalt, were determined using an Atomic Absorption Flame Emission Spectrophotometer (Perkin-Elmer Model AA-6200 from Shimadzu 7000, Japan) as reported by AOAC [39].

### 2.5. Cooking Characteristics of Roasted Camel Meat Burgers

Cooking yield and cooking loss percentages of camel meat burgers were calculated after roasting burger samples, according to Kim et al. [41]. Shrinkage (%) of camel meat burgers containing UBDF pulp was calculated after burger roasting as the diameter and thickness of the sample decreased, according to Wang et al. [42].

### 2.6. Instrumental Color Measurement

The measurement of (CIE) color values *L*^∗^ (lightness), *a*^∗^ (redness), and *b*^∗^ (yellowness) was conducted on the surface of the burger samples (raw or roasted) by using a Hunter Lab color measurement system (Hunter Lab, Color Flex, Hunter Associates Laboratory, USA) as described by Al-Juhaimi et al. [43].

### 2.7. Organoleptic Properties

Roasted burger samples were evaluated according to the method described by Rodríguez-Melcón et al. [44] with modifications. Ten panelists were chosen based on their previous experience and familiarity with the sensory analysis of meat products. Preparatory training sessions were provided to the panelists before the sensory evaluation to ensure that each panelist could identify and clarify each sensory attribute in cooked burgers. The burger samples were roasted at around 180°C for 3 min on each side using an electric grill (WA-BBQ 01, White Whale, China). The temperature of the center of the burgers reached approximately 80°C. The roasted burgers were kept warm and tested within 5-10 min after roasting. Panelists were asked to evaluate the following sensory parameters: appearance, color, odor, taste, tenderness, juiciness, and overall acceptability.

### 2.8. Statistical Analysis

All the measurements were done in triplicates, and data were presented as means ± SD. Analysis of variance (ANOVA, one-way analyses) was accompanied with Duncan's multiple range test for determining the significance at *p* < 0.05 level between means of treatments. According to Steel et al. [45], studies were carried out using SPSS software (version 23.0 for Windows, SPSS Inc., Chicago, USA).

## 3. Results

### 3.1. Proximate Chemical Composition and Mineral Content of Raw Materials

The proximate chemical composition and mineral contents of camel meat and UBDF pulp are presented in Table 2. The moisture, protein, fat, total carbohydrate, and ash contents of camel and UBDF pulp exhibited that camel meat is rich in protein and UBDF is rich in total carbohydrates. For mineral content, camel meat and UBDF presented valuable content of minerals, particularly in K. The UBDF was characterized by high total carbohydrates, fiber, and mineral contents, even higher than meat.

### 3.2. Proximate Chemical Composition and Mineral Content of Prepared Camel Burgers Incorporated with Different UBDF Pulp Levels

The chemical composition and mineral content results of prepared camel meat burgers with different levels of UBDF pulp either as fiber source or as fat replacer are shown in Table 3. Adding UBDF pulp as a fiber source significantly (*p* < 0.05) affected moisture, protein, fat, and fiber contents of differently prepared camel burger samples. On the other hand, ash content was not significantly affected (*p* > 0.05). Adding UBDF pulp significantly increased the K, Ca, and Mg contents while decreasing Fe and Cu contents. UBDF pulp levels increased (*p* < 0.05) the moisture content of the raw burgers from 58.32% in 0 pulp formula to 55.82% in 15% UBDF pulp formula. Also, it decreased the protein and fiber content significantly. No significant changes were noticed in its fat and ash contents. A nonsignificant increment of Zn and Co contents with increasing UBDF pulp levels as fiber source was noticed. In the case of adding UBDF pulp as a fat replacer in camel meat burgers, Table 3 reveals that different treatments' chemical compositions and mineral content are attributed to the amount of added UBDF pulp. Moisture content was significantly (*p* < 0.05) increased. The same trend was observed for ash and fiber content, while the content of protein was not significantly changed. A decrease in fat content and increased moisture, fiber, and carbohydrate contents were reported in uncooked samples. The K content increased significantly by increasing the levels of UBDF pulp as a fat replacer. In contrast, a nonsignificant increase was noticed for Ca, Zn, Fe, Mg, Cu, and Co contents with increasing UBDF pulp levels.

### 3.3. Cooking Characteristics of Camel Meat Burgers Incorporated with Different UBDF Pulp Levels

The cooking characteristics (shrinkage, cooking loss, and cooking yield) of roasted camel burgers incorporated with UBDF pulp as a fiber source (Table 4) or fat replacer are shown in Table 5. Incorporating UBDF pulp as fiber source or fat replacer to the camel burgers significantly (*p* < 0.05) improved the different cooking properties of the resultant burgers. An increase in the level of UBDF pulp was observed to increase the cooking yield and decrease the cooking loss and shrinkage percent of prepared burgers. Shrinkage of prepared camel burgers without adding UBDF pulp was significantly (*p* < 0.05) higher than that of burgers containing UBDF pulp as a fiber source or fat replacer. At the same time, by increasing the ratio of UBDF pulp, the shape and size of prepared camel meat burgers were improved during cooking.

### 3.4. Color Characteristics of Prepared Camel Meat Burger Incorporated with Different UBDF Pulp Levels

The color profile of raw and roasted camel burgers incorporated with UBDF pulp as fiber source is presented in Table 6 and as fat replacer is shown in Table 7. Table 6 shows that raw camel burgers containing different levels of UBDF pulp as a fiber source had a progressive increase in the lightness (*L*^∗^) values compared to the control burger sample. The same trend for redness (*a*^∗^) and yellowness (*b*^∗^) values was noticed in Table 6. The redness values of the camel meat burgers without adding UBDF pulp as a fiber source were the highest. On the other hand, as the ratios of UBDF pulp increased, the redness values of prepared burgers significantly (*p* < 0.05) decreased. Adding different levels of UBDF pulp as a fiber source gradually (*p* < 0.05) reduced the yellowness values of raw camel burgers compared to the control.

Regarding the color evaluation of roasted camel meat burgers containing UBDF pulp as a fiber source, Table 6 shows a significant increase in redness and yellowness values with increasing the addition levels. In addition, the lightness values of burger samples containing 15% UBDF pulp decreased considerably. The same results are recorded when adding UBDF pulp as fat replacer (Table 7), with little differences. The lightness and yellowness values did not significantly decrease with increasing UBDF pulp levels. In contrast, the decrement in redness values was significant. The color characteristics of roasting camel burgers containing UBDF pulp as a fat replacer could be noticed as a significant increase in *a*^∗^ and *b*^∗^ values while not significantly making the resultant burgers darker (decrease the *L*^∗^ values) (Table 7).

### 3.5. Sensory Characteristics of Camel Burgers Incorporated with Different UBDF Pulp Levels

The sensory evaluation of roasted camel burgers incorporating UBDF pulp as a fiber source is presented in Table 8, and the fat replacer is shown in Table 9. Camel meat burgers were evaluated for appearance, color, odor, taste, tenderness, juiciness, and overall acceptability. Incorporating UBDF pulp as a fiber source or fat replacer significantly increases all sensory attributes of the resultant camel burgers compared to the control. The burgers containing 15% UBDF pulp as fiber sources or those of 100% fat replacement by UBDF pulp recorded the highest scores in all sensory attributes and overall acceptability. It could be noticed that there are no significant differences between burger samples containing 7.5–15% UBDF pulp as a fiber source for all sensory attributes.

## 4. Discussion

The chemical composition of camel meat is essential to determine its nutritional value and approach meat during processing to different meat products. For instance, the moisture content of camel meat dictates the keeping and eating qualities of meat [46]. In contrast, protein and fat contents determine the manufacturing quality of meat [26]. The proximate chemical composition and mineral content agreed with the previous studies' differences in cuts, feeding, area, age, and sex [18, 19, 22, 46]. For example, the moisture content of camel meat decreases with increased animal age [18]. Comparative studies of the moisture content of camel meat with that of other species of the same age and sex found no species effects [18]. Slight differences exist between different meat cuts and meat from animals from different age groups [46]. Meats from young camels have similar protein content to those in young cattle, lamb, and goat meats [26, 47]. The fat content of camel meat ranged from 1.4 to 10.6%. The animal's age greatly affects the fat content, with camel meat from older animals containing more fat than meat from younger animals [22, 46]. Camel meats contain less fat than beef, lamb, and goat meats [18, 47]. The ash content of camel meat has been reported to be between 0.75 and 1.38%. Some reports suggested that the ash content varies with muscles and meat cuts [27, 47] and in meat from camel carcasses of different ages [18].

On the contrary, others found no effect of age and meat cut on ash content [46]. Camel meat has a relatively lower ash content than beef, lamb, and goat meat [18]. Elgasim and Alkanhal [47] reported that camel meat contained low Ca, Cu, Fe, K, and Zn contents. Kadim et al. [26] found lower Mg and K contents in camel meat than in cattle beef. Camel meat from animals grown in the desert had lower Ca, Fe, and K contents and higher Co, Cu, Mg, and Zn contents than farm animals [18, 47]. The UBDF pulp is characterized by high fiber content (19.60%). There are no available reports about the chemical composition of UBDF pulp which is also expected to differ depending on the processing or preparation method. The moisture, crude protein, lipid, ash, and total carbohydrate contents of Barhi fruits at the khalal maturity stage were reported as 83.2, 1.1, 0.1, 0.8, and 31.1%, respectively, on a fresh weight basis [38, 48].

Preparing camel meat burgers with varying amounts of UBDF pulp as a fiber source or a fat replacer revealed chemical and mineral content indicative of innovatively constructed goods with health advantages. The addition of UBDF pulp as a fiber source influenced various cooked camel burger samples' moisture, protein, and fiber concentrations. Furthermore, neither the fat nor the ash content changed noticeably. The addition of UBDF pulp resulted in a notable rise in K, Ca, Zn, Mg, and Co while a decrease in Fe and Cu levels. High total solids of UBDF pulp employed in this study may explain why camel burger samples of varying preparations reduced moisture content. Al-Juhaimi et al. [43] noticed the same but insignificant trend by adding moringa seed flour to beef burgers. Hawashin et al. [49] remarked that increasing the percentage of destoned olive cake powder in the patties significantly improved the protein and fat contents, cooking yield, moisture and fat retention, total phenolic, and DPPH radical scavenging activity. Al-Juhaimi et al. [50] indicated a considerable change in the chemical composition by adding Argel leaf powder (ALP) to camel meat patties. In the same context, Ammar [51] found that incorporating orange albedo powder at 5 and 10% as a fiber source in chicken meat nuggets significantly increased its fiber content compared to the control. No significant changes were noticed in its fat and ash contents. The increment of Zn and Co contents with increasing the levels of UBDF pulp as fiber source were nonsignificant [46–48].

The amount of pulp added was responsible for the product's chemical makeup and mineral content. There was a notable uptick in moisture, ash, and fiber content, whereas protein level was relatively unchanged. A high fiber content, like that found in the burgers we examined, can benefit human health in several ways [52]. Mansour and Khalil [53] replaced fat in the beef burger with hydrated wheat fiber (1 : 1) at 50, 100, and 150 g kg^−1^ fat. A decrease in fat content and increased moisture, protein, ash, and carbohydrate contents were reported in uncooked samples. Yilmaz and Daglioglu [54] replaced the fat of meatballs with different levels (5-20%) of oat bran. With increasing amounts of oat bran, the moisture and fat content decreased while protein and ash increased. Piñero et al. [15] studied the effect of oat's soluble fiber (*β*-glucan) as a fat replacer on low-fat beef patties' physical, chemical, microbiological, and sensory properties. They stated that significant (*p* < 0.05) improvements in cooking yield, fat retention, and low-fat patties' moisture were attributed to the water binding ability of *β*-glucan. Yasarlar et al. [55] revealed that moisture and fat content decreased with the addition of rye bran. With more rye bran, the amount of protein and ash increased. Our research found that using UBDF pulp as a fat substitute considerably increased K, Ca, and Mg levels.

Conversely, Zn and Co concentrations increased, but not by a statistically significant amount. Using UBDF pulp as a fat replacer resulted in a slight but nonsignificant decrease in Fe and Cu contents. Dates are rich in essential nutrients. They are a good source of iron, cobalt, zinc, and calcium and have above-average quantities of potassium and magnesium [38]. Hence, cooking characteristics such as shrinkage, cooking loss, and cooking yield of processed burgers are considerable quality attributes [56]. The cooking qualities of roasted camel burgers using UBDF pulp as a fiber source or fat replacer were examined. Incorporating UBDF pulp as a fiber source or fat replacer in camel burgers improved their cooking qualities. Increasing the level of UBDF pulp increases cooking yield and decreases cooking loss and shrinkage [14, 15, 25, 49]. These results could be due to the increased ability of UBDF pulp to retain water and fat in camel burger samples. Al-Juhaimi et al. [50] noticed the same results. Adding ALP to camel meat patties enhanced cooking yield and shrinkage compared to control patties [50]. Water retention and fat binding in the protein matrix affect cooking yield and meat product structure. Similar experiments found that UBDF pulp's ability to retain moisture in the burger matrix improved cooking [14, 15, 25, 49].

During cooking, protein denaturation, water evaporation, and loss of melted fat and meat fluids cause camel burgers to shrink. The shrinkage of camel burgers without UBDF pulp was much higher than that of burgers with it as a fiber source or fat replacer. Increasing the UBDF pulp ratio increased camel meat burgers' form and size during cooking [25, 43]. The reduced shrinkage noticed in the UBDF pulp formulated burgers could be due to the binding ability of UBDF pulp, which kept the functional properties of the protein in prepared burgers and improved its ability to retain moisture and melted fat during the cooking step. Hawashin et al. [49] and Al-Juhaimi et al. [50] noticed that adding destoned olive cake to beef patties reduces shrinkage and improves cooking yield, moisture and fat retention, and phenolic and radical scavenging activity.

Camel burgers made with UBDF pulp were evaluated for their color characteristics before and after cooking. However, the cooked raw camel burgers showed a progressive rise in lightness (*L*^∗^) values compared to the control burger sample. This may be because of the lightness of the burger samples and the yellowish tint of UBDF pulp as a fiber source [57]. On the contrary, no significant changes in lightness values of a beef burger containing pea fiber as a fiber source were observed [14]. Hawashin et al. [43] observed a reduction of *L*^∗^ values in raw beef patties formulated with destoned olive cake powder. Also, Al-Juhaimi et al. [43] reported that incorporating moringa seeds significantly decreased the *L*^∗^ values of beef patties. Yilmaz and Daglioglu [54] observed that the lightness of meatballs increased as the amount of oat bran increased during preparing meatball samples. The redness values of the camel meat burgers without adding UBDF pulp as a fiber source were the highest. As the ratios of UBDF pulp increased, the redness values of prepared burgers significantly decreased, which was agreed upon [43, 49, 50]. Simultaneously, the lightness of prepared camel burger samples could be due to adding fiber and reducing the myoglobin content [14]. Adding UBDF pulp as a fiber source gradually decreased the yellowness values of raw camel burgers compared to control samples. Camel meat burgers containing UBDF pulp as a fiber source showed a significant increase in redness and yellowness values with increased addition levels. In addition, burger samples containing 15% UBDF pulp showed a significant decrease in lightness values. These results may be due to the high content of carbohydrates in UBDF pulp, which led to the Maillard reaction during the cooking process of low-fat and high-fiber camel meat burgers. In contrast, the redness values decreased significantly related to increasing adding levels. Ammar et al. [58] found no difference in *L*^∗^ values between meatballs without or with pumpkin flour as a fat replacer. However, the *a*^∗^ and *b*^∗^ values of prepared meatball samples with pumpkin flour as fat replacers were dramatically influenced. The *a*^∗^ and *b*^∗^ values of roasted camel burgers with UBDF pulp increased dramatically, but *L*^∗^ values decreased slightly. It could be noticed that instrumental color change is affected by added material color and content of Maillard reaction required components [58].

It is well known that the acceptability of meat products is flavor, color, appearance, tenderness, and juiciness dependent. However, the texture (tenderness) and juiciness of meat products are crucial sensory characteristics that influence consumers' palatability of meat products [59]. The sensory evaluation of roasted camel burgers incorporated with UBDF pulp indicated a significant increase in burger attributes compared to control burgers. Burgers containing 15% UBDF pulp (as fiber source) or those of 100% fat replacement by UBDF pulp recorded the highest scores in all sensory attributes and overall acceptability. It could be noticed that there are no significant differences between burger samples containing 7.5–15% UBDF pulp. As a result, it is safe to say that UBDF pulp, within the range of concentrations studied, can be utilized as a novel fiber source or fat replacer in the preparation of camel meat burgers without altering the product's sensory qualities. Al-Juhaimi et al. [50] observed a significant decrease in color and taste attributes of camel patties containing ALP at higher concentrations (4 and 6%) compared to the control or 2% ALP-formulated patties. Also, Al-Juhaimi et al. [49] reported an insignificant decrease in the sensory scores of cooked beef patties with high moringa seed flour levels. Eldemery [60] found that beef burgers' sensory characteristics and overall acceptability were improved by using 5% of raw or cooked orange albedo. Ammar et al. [58] observed an insignificant difference in sensory properties between the control and prepared meatball samples containing date seed powder, wheat germ, or pumpkin flour. Dawkins et al. [61] showed that the sensory panel showed no significant difference in tenderness, juiciness, beef flavor intensity, and overall palatability at the different added fiber levels. According to Kenawi et al. [62], the product containing 5% low-fat soy flour or mung bean powder had the highest scores for all studied sensory attributes and overall acceptability. Verma et al. [63] incorporated different dietary fiber sources, namely, pea hull, apple pulp, gram hull, and bottle gourd, in various combinations in low-fat chicken nuggets. There were differences in different quality attributes among treatments, but organoleptically, the latter was comparable to the base control samples. Viuda-Martos et al. [31] also reported decreased juiciness and increased hardness perception on sensory analysis of mortadella added with orange dietary fiber. However, low-fat patties were found to be of lower degree of likeness in taste but juicer than the control (*p* < 0.05). Besides appearance, tenderness and color were not affected by the addition of oat's soluble fiber. Oat fiber can be used successfully as a fat substitute in low-fat beef patties, as recommended by Piñero et al. [15].

## 5. Conclusions

The UBDF contained a high amount of total carbohydrates, fiber, and minerals. It has a high fiber content (19.60%) and a low-fat content. Incorporating varying levels of UBDF pulp as a fiber source or fat replacer in camel burgers improved fiber contents, cooking properties, and surface color characteristics without compromising organoleptic quality. The results demonstrated that adding UBDF as a fiber source decreased the moisture and protein content while increasing the total carbs, including fiber. In camel meat burgers, K and Ca levels were significantly increased in UBDF level-dependent manure. Adding UBDF as a fat replacer increased moisture and total carbohydrate content while decreasing fat content significantly. Roasted camel burger cooking parameters like shrinkage, cooking loss, and cooking yield were enhanced in level-dependent manure. Camel burgers with UBDF pulp added had significantly higher lightness (*L*^∗^) ratings and lower redness (*a*^∗^) and yellowness (*b*^∗^). The organoleptic qualities of camel burgers made with varying amounts of UBDF pulp were superior to those of camel burgers made with regular camel meat. Overall acceptance was higher when UBDF was added as a fiber source, between 7.5% and 15%, compared to the control samples. In the same context, adding UBDF as a fat replacer at a 100% replacement of added fats resulted in overall acceptability compared to the control sample. Accordingly, the use of UBDF pulp as a novel fiber source or fat replacer is in keeping with customer tastes and the trend toward food products, including healthier nutritional elements. The results of this study indicate that UBDF pulp has the potential to be employed as a functional addition in the creation of high-fiber and/or low-fat camel meat burgers.

## Figures and Tables

**Table 1:** Ingredients (%) of camel meat burger blends formulated with UBDF pulp.

Ingredients (%)	Control	UBDF pulp as fiber source (%)					UBDF pulp as fat replacer (%)		
		2.5	5.0	7.5	10.0	15.0	50	75	100
Camel meat	65.0	62.5	60.0	57.5	55.0	50.0	65.0	65.0	65.0
Camel-back fat	15.0	15.0	15.0	15.0	15.0	15.0	7.5	3.75	0.0
Starch	7.5	7.5	7.5	7.5	7.5	7.5	7.5	7.5	7.5
Water as ice	10.0	10.0	10.0	10.0	10.0	10.0	10.0	10.0	10.0
Spices	2.5	2.5	2.5	2.5	2.5	2.5	2.5	2.5	2.5
Unpollinated Barhi date fruits pulp	0.0	2.5	5.0	7.5	10.0	15.0	7.5	11.25	15.0

**Table 2:** Chemical composition and mineral content of raw materials.

Materials	Chemical composition (g 100 g^−1^ fw)						
	Moisture	Protein	Fat	Ash	Total carbohydrates^∗^		Fiber
Camel meat	73.89 ± 0.70	20.11 ± 1.00	2.63 ± 0.40	1.01 ± 0.03	2.36 ± 0.60		—
UBDF pulp	57.15 ± 0.82	1.65 ± 0.20	0.10 ± 0.01	1.20 ± 0.05	39.90 ± 1.10		19.60 ± 0.51

Materials	Mineral content (mg 100 g^−1^ fw)						
	K	Ca	Zn	Fe	Mg	Cu	Co
Camel meat	228.00 ± 6.50	5.50 ± 0.33	3.20 ± 0.40	1.90 ± 0.02	17.70 ± 2.60	0.20 ± 0.01	0.003 ± 0.00
UBDF pulp	759.40 ± 8.30	71.60 ± 2.11	12.40 ± 0.20	0.07 ± 0.06	41.50 ± 5.00	0.13 ± 0.01	0.015 ± 0.00

^∗^Calculated by the difference.

**Table 3:** Chemical composition and mineral content of camel meat burger formulas prepared with different levels of UBDF pulp as a fiber source and fat replacer.

Parameters	Burger contained UBDF pulp as a fiber source (g 100 g^−1^)						Burger contained UBDF pulp as fat replacer (g 100 g^−1^)		
	0.0	2.5	5.0	7.5	10.0	15.0	50	75	100
Moisture	58.32^a^ ± 0.11	57.91^b^ ± 0.13	57.49^c^ ± 0.10	57.05^d^ ± 0.08	56.66^e^ ± 0.10	55.82^f^ ± 0.10	62.61^c^ ± 0.20	64.76^b^ ± 0.40	66.90^a^ ± 0.33
Protein	13.82^a^ ± 0.37	13.26^b^ ± 0.20	12.80^c^ ± 0.18	12.34^d^ ± 0.03	11.88^e^ ± 0.05	11.05^f^ ± 0.08	13.84^a^ ± 0.30	13.91^a^ ± 0.25	13.97^a^ ± 0.31
Fat	16.86^a^ ± 0.06	16.79^ab^ ± 0.09	16.73^ab^ ± 0.10	16.66^b^ ± 0.11	16.61^b^ ± 0.10	16.48^b^ ± 0.10	9.38^a^ ± 0.15	5.62^b^ ± 0.20	1.88^c^ ± 0.12
Ash	0.97^a^ ± 0.03	0.97^a^ ± 0.05	0.97^a^ ± 0.06	0.97^a^ ± 0.05	0.98^a^ ± 0.05	1.00^a^ ± 0.02	1.06^a^ ± 0.06	1.11^a^ ± 0.03	1.15^a^ ± 0.02
Total carbohydrates	10.03^f^ ± 0.09	11.07^e^ ± 0.13	12.01^d^ ± 0.12	12.98^c^ ± 0.21	13.87^b^ ± 0.19	15.65^a^ ± 0.09	13.11^c^ ± 0.16	14.60^b^ ± 0.23	16.10^a^ ± 0.32
Fiber	0.27^f^ ± 0.02	0.76^e^ ± 0.06	1.28^d^ ± 0.03	1.74^c^ ± 0.04	2.23^b^ ± 0.02	3.21^a^ ± 0.03	1.74^c^ ± 0.05	2.48^b^ ± 0.18	3.22^a^ ± 0.16

Mineral content (mg 100 g^−1^)									
K	0.0	2.5	5.0	7.5	10.0	15.0	50	75	100
	682.10^e^ ± 9.30	737.70^d^ ± 11.55	768.70^bc^ ± 10.12	794.80^b^ ± 15.44	803.00^ab^ ± 28.13	850.00^a^ ± 22.19	771.40^b^ ± 14.88	833.30^a^ ± 26.19	862.60^a^ ± 19.13
Ca	22.40^d^ ± 1.81	26.50^c^ ± 2.05	29.30^bc^ ± 1.99	33.20^b^ ± 2.44	37.10^ab^ ± 3.01	38.80^a^ ± 2.25	38.80^a^ ± 1.89	40.00^a^ ± 2.11	42.50^a^ ± 2.09
Zn	9.20^a^ ± 1.23	9.70^a^ ± 1.73	10.00^a^ ± 1.62	10.50^a^ ± 1.55	11.00^a^ ± 1.89	11.30^a^ ± 1.22	10.40^a^ ± 1.99	11.30^a^ ± 1.23	11.40^a^ ± 0.88
Fe	0.91^a^ ± 0.05	0.83^ab^ ± 0.09	0.82^b^ ± 0.03	0.81^b^ ± 0.11	0.78^bc^ ± 0.12	0.72^c^ ± 0.09	0.77^a^ ± 0.06	0.76^a^ ± 0.05	0.72^a^ ± 0.08
Mg	10.93^b^ ± 1.05	11.34^b^ ± 1.18	12.97^ab^ ± 1.99	13.23^a^ ± 2.31	13.88^a^ ± 1.07	14.11^a^ ± 1.28	11.57^a^ ± 1.56	12.91^a^ ± 1.87	13.46^a^ ± 2.13
Cu	0.18^a^ ± 0.01	0.17^ab^ ± 0.04	0.14^ab^ ± 0.04	0.13^ab^ ± 0.05	0.13^ab^ ± 0.07	0.12^b^ ± 0.04	0.13^a^ ± 0.06	0.12^a^ ± 0.04	0.11^a^ ± 0.01
Co	0.010^a^ ± 0.002	0.011^a^ ± 0.004	0.012^a^ ± 0.003	0.013^a^ ± 0.003	0.014^a^ ± 0.004	0.014^a^ ± 0.003	0.012^a^ ± 0.005	0.013^a^ ± 0.003	0.014^a^ ± 0.002

^a,b,c^No significant difference (*p* > 0.05) between any two means within the same row with the same superscripted letters.

**Table 4:** Cooking characteristics of roasted camel meat burger as affected by adding various levels of UBDF as a fiber source.

Burger samples^∗^	Cooking characteristics (%)		
	Shrinkage	Cooking loss	Cooking yield
0.0%	26.67^a^ ± 1.12	36.98^a^ ± 1.45	63.02^c^ ± 1.45
2.5%	21.67^ab^ ± 0.53	35.46^ab^ ± 0.66	64.54^bc^ ± 0.66
5.0%	21.67^ab^ ± 0.18	34.27^b^ ± 0.31	65.73^b^ ± 0.31
7.5%	16.67^bc^ ± 0.27	33.82^bc^ ± 0.45	66.52^ab^ ± 0.45
10.0%	10.00^c^ ± 0.22	31.76^c^ ± 0.60	68.24^a^ ± 0.60
15.0%	9.17^c^ ± 0.22	31.76^c^ ± 0.41	68.24^a^ ± 0.41

^∗^Camel meat burgers partially substituted with UBDF pulp as a fiber source. ^a,b,c^No significant difference (*p* > 0.05) between any two means within the same column with the same superscripted letters.

**Table 5:** Cooking characteristics of roasted camel meat burger as affected by replacing fat with different levels of UBDF pulp.

Burger samples^∗^	Cooking characteristics (%)		
	Shrinkage	Cooking loss	Cooking yield
0%	26.67^a^ ± 1.12	36.98^a^ ± 1.45	63.02^c^ ± 1.45
50%	16.67^b^ ± 0.23	33.56^b^ ± 0.42	66.44^b^ ± 0.42
75%	14.67^b^ ± 0.22	33.22^b^ ± 0.45	66.78^b^ ± 0.45
100%	13.17^b^ ± 0.19	28.43^c^ ± 0.33	71.57^a^ ± 0.33

^∗^Camel meat burgers partially substituted with UBDF pulp as a fat replacer. ^a,b,c^No significant difference (*p* > 0.05) between any two means within the same column with the same superscripted letters.

**Table 6:** Instrumental color characteristics of uncooked and roasted camel meat burger as affected by adding various levels (%) of UBDF as a fiber source.

Burger samples^∗^	Uncooked camel meat burger			Roasted camel meat burger		
	*L* ^∗^	*a* ^∗^	*b* ^∗^	*L* ^∗^	*a* ^∗^	*b* ^∗^
0.0%	33.80^b^ ± 0.89	2.74^a^ ± 0.08	8.88^a^ ± 0.12	33.12^b^ ± 0.95	3.08^c^ ± 0.16	9.12^b^ ± 0.04
2.5%	35.68^ab^ ± 0.65	2.51^b^ ± 0.06	8.54^b^ ± 0.09	34.65^ab^ ± 0.64	3.05^c^ ± 0.11	9.24^b^ ± 0.07
5.0%	36.55^a^ ± 0.64	2.46^bc^ ± 0.05	8.32^bc^ ± 0.11	35.51^a^ ± 0.65	2.99^c^ ± 0.13	9.28^b^ ± 0.04
7.5%	36.93^a^ ± 0.62	2.40^bc^ ± 0.06	8.23^bc^ ± 0.13	36.06^a^ ± 0.52	3.45^b^ ± 0.16	9.11^b^ ± 0.10
10.0%	37.26^a^ ± 0.63	2.22^cd^ ± 0.06	8.05^cd^ ± 0.64	35.56^a^ ± 0.64	3.98^a^ ± 0.09	9.96^a^ ± 0.12
15.0%	38.06^a^ ± 0.68	2.11^d^ ± 0.14	7.85^d^ ± 0.13	30.80^c^ ± 0.46	4.11^a^ ± 0.16	10.35^a^ ± 0.05

^∗^Camel meat burgers partially substituted with UBDF pulp as a fiber source, *L*^∗^ value is a measure of lightness ranging from 0 (black) to 100 (white), *a*^∗^ value ranges from -100 (greenness) to +100 (redness), and *b*^∗^ value ranges from -100 (blueness) to +100 (yellowness). ^a,b,c^No significant difference (*p* > 0.05) between any two means within the same column with the same superscripted letters.

**Table 7:** Instrumental color characteristics of uncooked and roasted camel meat burger as affected by replacing fat with different levels (%) of UBDF pulp.

Burger samples^∗^	Uncooked camel meat burger			Roasted camel meat burger		
	*L* ^∗^	*a* ^∗^	*b* ^∗^	*L* ^∗^	*a* ^∗^	*b* ^∗^
0%	33.81^a^ ± 0.89	2.75^a^ ± 0.08	8.88^a^ ± 0.12	33.12^a^ ± 0.94	3.09^d^ ± 0.04	9.12^b^ ± 0.16
50%	35.47^a^ ± 0.69	2.63^ab^ ± 0.08	8.84^a^ ± 0.11	32.64^a^ ± 0.95	3.56^c^ ± 0.05	9.41^b^ ± 0.13
75%	35.67^a^ ± 0.68	2.50^b^ ± 0.06	8.77^a^ ± 0.11	31.81^a^ ± 1.01	3.83^b^ ± 0.04	9.77^a^ ± 0.08
100%	35.74^a^ ± 0.68	2.45^b^ ± 0.05	8.64^a^ ± 0.10	31.01^a^ ± 0.92	4.13^a^ ± 0.07	10.07^a^ ± 0.06

^∗^Camel meat burgers partially substituted with UBDF pulp as a fat replacer, *L*^∗^ value is a measure of lightness ranging from 0 (black) to 100 (white), *a*^∗^ value ranges from -100 (greenness) to +100 (redness), and *b*^∗^ value ranges from -100 (blueness) to +100 (yellowness). ^a,b,c^No significant difference (*p* > 0.05) between any two means within the same column with the same superscripted letters.

**Table 8:** Sensory characteristics of roasted camel meat burger as affected by adding various levels (%) of UBDF as a fiber source.

Burger samples	Sensory characteristics						
	Appearance	Color	Odor	Taste	Tenderness	Juiciness	Overall acceptability
0.0%	12.8^b^ ± 0.53	12.8^b^ ± 0.49	12.2^c^ ± 0.44	13.2^b^ ± 0.53	14.9^c^ ± 0.67	15.8^b^ ± 0.44	81.7^b^ ± 2.33
2.5%	13.4^ab^ ± 0.49	13.3^ab^ ± 0.42	12.9^bc^ ± 0.50	13.2^b^ ± 0.42	15.2^bc^ ± 0.48	16.3^b^ ± 0.59	84.4^b^ ± 2.17
5.0%	13.6^ab^ ± 0.37	13.7^ab^ ± 0.33	12.8^bc^ ± 0.44	12.9^b^ ± 0.31	16.8^ab^ ± 0.71	16.3^b^ ± 0.68	86.0^b^ ± 2.06
7.5%	14.2^a^ ± 0.20	14.2^a^ ± 0.25	13.7^ab^ ± 0.26	13.9^ab^ ± 0.18	17.3^a^ ± 0.70	18.0^a^ ± 0.42	91.4^a^ ± 1.27
10.0%	14.1^a^ ± 0.18	14.3^a^ ± 0.21	14.4^a^ ± 0.22	14.3^a^ ± 0.21	17.9^a^ ± 0.67	19.0^a^ ± 0.29	93.9^a^ ± 1.06
15.0%	13.8^ab^ ± 0.29	14.2^a^ ± 0.13	14.4^a^ ± 0.16	14.3^a^ ± 0.15	18.2^a^ ± 0.44	19.0^a^ ± 0.30	93.9^a^ ± 1.60

^∗^Camel meat burgers partially substituted with UBDF pulp as a fiber source. ^a,b,c^No significant difference (*p* > 0.05) between any two means within the same column with the same superscripted letters.

**Table 9:** Sensory characteristics of roasted camel meat burger affected by replacing fat with different levels (%) of UBDF pulp.

Burger samples^∗^	Sensory characteristics						
	Appearance	Color	Odor	Taste	Tenderness	Juiciness	Overall acceptability
0%	12.3^c^ ± 0.39	11.0^c^ ± 0.36	10.8^c^ ± 0.49	11.1^c^ ± 0.64	11.3^d^ ± 0.71	11.2^c^ ± 0.94	67.4^c^ ± 3.07
50%	13.5^b^ ± 0.22	13.0^b^ ± 0.33	12.7^b^ ± 0.47	12.4^bc^ ± 0.56	14.0^c^ ± 0.36	14.9^b^ ± 0.67	80.5^b^ ± 2.20
75%	14.0^ab^ ± 0.15	13.9^a^ ± 0.18	13.4^ab^ ± 0.22	13.5^ab^ ± 0.37	16.1^b^ ± 0.48	16.6^b^ ± 0.22	84.5^b^ ± 1.33
100%	14.4^a^ ± 0.16	14.3^a^ ± 0.15	14.4^a^ ± 0.16	14.5^a^ ± 0.17	18.3^a^ ± 0.36	18.8^a^ ± 0.25	93.60^a^ ± 1.26

^∗^Camel meat burgers partially substituted with UBDF pulp as a fat replacer. ^a,b,c^No significant difference (*p* > 0.05) between any two means within the same column with the same superscripted letters.

## Data Availability

The data used to support the findings of this study are available from the corresponding author upon request.
